# Impact of the polygenic risk scores for attention‐deficit/hyperactivity disorder in Alzheimer's disease

**DOI:** 10.1002/alz.70003

**Published:** 2025-02-25

**Authors:** Douglas T. Leffa, Guilherme Povala, Bruna Bellaver, João Pedro Ferrari‐Souza, Pamela C. L. Ferreira, Firoza Z. Lussier, Cristiano Schaffer Aguzzoli, Carolina Soares, Hussein Zalzale, Francieli Rohden, Guilherme Bauer‐Negrini, Sarah Abbas, Maitê Schneider, Joseph Therriault, Oscar L. Lopez, Victor L. Villemagne, William E. Klunk, Dana L. Tudorascu, Ann D. Cohen, Pedro Rosa‐Neto, Eduardo R. Zimmer, Thomas K. Karikari, Luis Augusto Rohde, Brooke S. G. Molina, Tharick A. Pascoal, the Alzheimer's Disease Neuroimaging Initiative

**Affiliations:** ^1^ Department of Psychiatry University of Pittsburgh Pittsburgh Pennsylvania USA; ^2^ Graduate Program in Biological Sciences: Biochemistry Universidade Federal do Rio Grande do Sul Porto Alegre RS Brazil; ^3^ Brain Institute of Rio Grande do Sul Pontifical Catholic University of Rio Grande do Sul Porto Alegre RS Brazil; ^4^ ADHD Outpatient Program & Development Psychiatry Program Hospital de Clínicas de Porto Alegre, Universidade Federal do Rio Grande do Sul Porto Alegre RS Brazil; ^5^ Translational Neuroimaging Laboratory, McGill University Rue University Montréal QC Canada; ^6^ Department of Neurology University of Pittsburgh Pittsburgh Pennsylvania USA; ^7^ Graduate Program in Biological Sciences: Pharmacology and Therapeutics Department of Pharmacology Universidade Federal do Rio Grande do Sul Porto Alegre RS Brazil; ^8^ Department of Psychiatry and Neurochemistry Institute of Neuroscience and Physiology The Sahlgrenska Academy University of Gothenburg Gothenburg Sweden; ^9^ National Institute of Developmental Psychiatry & National Center for Innovation and Research in Mental Health (CISM) Porto Alegre RS Brazil; ^10^ Medical Council Centro Universitário de Jaguariúna (UNIFAJ) Jaguariúna SP Brazil; ^11^ Medical Council Centro Universitário Max Planck (UNIMAX) Indaiatuba SP Brazil; ^12^ Departments of Psychiatry Psychology, Pediatrics Clinical and Translational Science University of Pittsburgh Pittsburgh Pennsylvania USA

**Keywords:** ADHD, Alzheimer's disease, amyloid, cognition, dementia, executive function, glucose hypometabolism, mild cognitive impairment, PET, phosphorylated tau, polygenic risk scores

## Abstract

**INTRODUCTION:**

Epidemiological studies indicate a link between attention‐deficit/hyperactivity disorder (ADHD) and elevated risk of dementia. However, the impact of ADHD on cognition and Alzheimer's disease (AD) biomarkers in individuals with cognitive impairment remains unclear.

**METHODS:**

We computed weighted ADHD polygenic risk scores (ADHD‐PRS) in 938 cognitively impaired participants (674 mild cognitive impairment [MCI] and 264 dementia; mean age 73.5 years). A subset underwent cerebrospinal fluid (CSF) analysis for amyloid beta (Aβ) and phosphorylated tau, as well as fluorodeoxyglucose positron emission tomography ([^18^F]FDG‐PET).

**RESULTS:**

We observed lower executive function in individuals with high ADHD‐PRS for both MCI and dementia participants. Higher levels of CSF phosphorylated tau, but not Aβ, were observed in dementia participants with higher ADHD‐PRS. Increased ADHD‐PRS was associated with glucose hypometabolism in the frontal and parietal cortices.

**DISCUSSION:**

ADHD‐PRS is associated with a more severe disease presentation in individuals with cognitive impairment due to dementia, characterized by impaired executive function, elevated tau pathology, and hypometabolism in the frontal and parietal cortices.

**Highlights:**

- We calculated the genetic liability for attention‐deficit/hyperactivity disorder (ADHD) using polygenic risk scores (ADHD‐PRS).
- Elevated ADHD‐PRS was associated with executive function deficits in individuals with mild cognitive impairment (MCI) or Alzheimer's disease (AD) dementia.
- Higher levels of cerebrospinal fluid (CSF) phosphorylated tau, but not amyloid beta (Aβ), were observed in dementia participants with higher ADHD‐PRS.
- Higher ADHD‐PRS was associated with brain hypometabolism in individuals with AD dementia.
- Hypometabolism in the parietal cortex mediated the effects of ADHD‐PRS on executive function.

## 1 BACKGROUND

Attention‐deficit/hyperactivity disorder (ADHD) is a neurodevelopmental disorder characterized by persistent symptoms of inattention and/or hyperactivity/impulsivity.^1^ It is highly heritable,^2^ with a prevalence of 5.3% among school‐aged children,^3^ 2.5% among adults,^4^ and 2.1% among adults 50 year of age or older.^5^ In recent years, there has been a notable increase in interest in identifying and understanding ADHD in the older adult population.^6^ Moreover, given the expanded population of adults age 65+, it is anticipated that the overall count of individuals 50 years and older meeting the diagnostic criteria for ADHD will inevitably rise.^6^ Consequently, comprehending the link between this disorder and common age‐related diseases has become an urgent concern.

Of particular significance is the relationship between ADHD and age‐related cognitive impairment. Large‐scale epidemiological studies utilizing register data from millions of individuals indicated an increased likelihood of a diagnosis of MCI and dementia (such as AD dementia) among individuals with ADHD.^7^, ^8^, ^9^, ^10^ The pathophysiology of AD involves the aggregation of amyloid beta (Aβ) in extracellular neuritic plaques, followed by the accumulation of hyperphosphorylated tau (p‐tau) in cell bodies and dendrites as neurofibrillary tangles, which is closely linked to neurodegeneration and cognitive decline.^11^ Although the underlying mechanisms linking ADHD and AD remain unclear, recent studies suggest that individuals with ADHD may have reduced resilience to Aβ pathology, leading to a decline in cognition at lower pathological levels. ^12^, ^13^ In cognitively unimpaired individuals, a higher genetic risk for ADHD was associated with a progressive rise in brain tau pathology alongside frontoparietal atrophy.^13^ However, the extent to which ADHD is associated with cognitive dysfunction and markers of AD pathology beyond what is anticipated in MCI and AD individuals remains unclear.

One of the challenges in studying the association between ADHD and AD dementia is the absence of large studies evaluating cognitive function and AD biomarkers in older adults with a clinical diagnosis of ADHD. Therefore, we explored this association using a well‐established biomarker of ADHD, the ADHD polygenic risk score (ADHD‐PRS), which represents the combined genetic liability for the disorder and is highly associated with ADHD diagnosis and related traits in independent samples.^14^ Here, we tested the following hypotheses: (1) ADHD‐PRS is associated with worsening cognitive function in patients with MCI and AD; (2) ADHD‐PRS correlates with elevated biomarkers of AD pathology within this population, including CSF levels of Aβ and p‐tau, and fluorodeoxyglucose positron emission tomography ([^18^F]FDG‐PET); and (3) lower cognitive function linked with ADHD‐PRS is mediated by brain glucose hypometabolism. In addition, we tested whether the findings were specific for ADHD by exploring the association between both cognitive function and AD biomarkers with the genetic risk for prevalent psychiatric disorders (schizophrenia [SCZ], bipolar disorder [BD], major depressive disorder [MDD], and autism spectrum disorder [ASD]).

## 2 METHODS

### 2.1 Participants

We used data from the Alzheimer's Disease Neuroimaging Initiative (ADNI), a multicenter study aimed at developing clinical, imaging, genetic, and biochemical biomarkers for the early detection and tracking of AD (http://adni.loni.usc.edu; for more detail, refer to previous publications^15^). ADNI's inclusion criteria relevant for this study encompassed age between 55 and 90, absence of MDD or BD (Diagnostic and Statistical Manual of Mental Disorders, Fourth Edition [DSM‐IV] criteria) within the past year, no history of SCZ (DSM‐IV criteria), and a Geriatric Depression Scale (GDS) score lower than 6.^15^ A comprehensive list of ADNI's enrollment criteria is available elsewhere.^15^ Data were downloaded from the ADNI data repository in December 2022. The institutional review boards of all participating sites approved the ADNI study, and all research participants or their authorized representatives provided written informed consent.

**RESEARCH IN CONTEXT**

1. **Systematic review**: The authors reviewed the literature using traditional (e.g., PubMed) sources. Although the literature exploring the relationship between attention‐deficit/hyperactivity disorder (ADHD) and age‐related cognitive impairment remains scarce, there has been a recent increase in publications on this topic. The relevant citations are appropriately cited.
2. **Interpretation**: Our results suggest that older participants with dementia due to Alzheimer's disease and greater genetic susceptibility to ADHD have a more severe presentation of the disease, characterized by impaired executive function, elevated tau pathology, and hypometabolism in the frontal and parietal cortices.
3. **Future directions**: Future studies should focus on: (1) validating these results in randomly sampled populations to avoid sampling bias; (2) further investigating potential confounders in the association between ADHD and cognitive decline; and (3) exploring cognitive function and Alzheimer's disease biomarkers in individuals diagnosed with ADHD.

We studied MCI and AD participants from ADNI who had available whole‐genome information and underwent a baseline clinical assessment with neuropsychological testing. Genotype data were available from 1674 individuals. As described previously, 350 participants were excluded after the genotype data quality control.^16^ Among the 1324 remaining individuals, 385 were cognitively unimpaired and, consequently, were excluded. One individual did not have a full clinical assessment. Finally, 938 participants were included in our analyses. Among these, 696 participants had baseline CSF levels of Aβ and p‐tau, and 663 had baseline [^18^F]FDG‐PET data. Similar demographic and clinical characteristics were observed between individuals with and without CSF data (Table S1).

### 2.2 Polygenic risk score

The genotyping, quality control, and imputation of the genetic data from the ADNI sample have been described previously.^16^ We used PRSice software v2.2^17^ to calculate the PRSs from genome‐wide association studies of ADHD, AD, SCZ, BD, MDD, and ASD. For additional methodological information, refer to the Supplementary Material.

### 2.3 Cognitive function

Each participant underwent a comprehensive clinical and neuropsychological assessment at baseline. As a primary outcome, we used a validated composite score for executive function, memory, and language.^18^, ^19^, ^20^ Details about each composite score can be found in the Supplementary Material.

### 2.4 Cerebrospinal fluid (CSF) biomarkers

CSF Aβ1‐42 (Aβ1‐42) and tau phosphorylated at threonine 181 (p‐tau181) were quantified using fully automated Elecsys immunoassays (Roche Diagnostics). Measurements outside the analytical range were managed as described in the Supplementary Material. Aβ positivity was defined as CSF Aβ1‐42 <976.6 pg/mL, and tau positivity was defined as p‐tau181 >24 pg/mL, as described previously.^21^

### 2.5 MRI acquisition and processing

We acquired pre‐processed 3T T1‐weighted magnetic resonance imaging (MRI) scans following the established ADNI acquisition protocols. For complete details of the ADNI neuroimaging data acquisition protocol and pre‐processing of MRI data, visit http://adni.loni.usc.edu/methods/mri‐tool/mri‐analysis/. We employed advanced normalization tools (ANTs) to perform linear and non‐linear registration procedures, aligning the images with the ADNI template space.^22^ We conducted visual inspections on all images to confirm precise alignment with the ADNI template.

### 2.6 Positron emission tomography (PET) acquisition and processing

We downloaded pre‐processed [^18^F]FDG‐PET images from the ADNI database. For comprehensive details regarding the ADNI neuroimaging data acquisition protocol and PET data pre‐processing, visit http://adni.loni.usc.edu/methods/pet‐analysis‐method/pet‐analysis/. The images underwent spatial normalization to be co‐registered to the ADNI standardized space. First, we applied an automated registration process to align PET images with their corresponding T1‐weighted image space. In addition, we applied linear and non‐linear transformations derived from the T1‐weighted image space to the ADNI template space. All transformations were executed using ANTs. Subsequently, we spatially smoothed the PET images to achieve a final resolution of 8 mm full width at half maximum (FWHM). We then conducted visual inspections to ensure precise alignment with the ADNI template. Standardized uptake value ratio (SUVr) maps were derived for [^18^F]FDG‐PET using the pons as the reference region.

### 2.7 Statistical analyses

The associations between PRS (transformed into *z*‐scores and categorized into low and high [<0 and >0, respectively]) and cognitive function (transformed into *z*‐scores) were examined with linear regression models adjusting for sex assigned at birth, age, and ancestry (using the first seven principal components, as previously performed for ADNI datasets ^16^). Sensitivity analyses were carried out to explore the role of potential confounders such as genetic risk for AD, vascular risk factors (VRFs), obesity (using body mass index [BMI]), and educational attainment (using years of education). VRF burden was assessed using a composite score, and a score equal to or higher than two was defined as elevated^13^, ^23^ (for details, refer to the Supplementary Material).

Differences in CSF Aβ1‐42 and p‐tau181 between low and high PRS scores were examined using linear regression models adjusted for sex, age, and ancestry. For those analyses, CSF Aβ1‐42 and p‐tau181 were transformed into *z*‐scores. All analyses were performed for MCI and dementia participants separately.

We employed voxel‐wise linear regression models to examine the relationships between [^18^F]FDG‐PET metabolism and ADHD‐PRS. These models were adjusted for age, sex, and ancestry. Mediation analyses were performed using structural equation modeling (SEM) with maximum likelihood including missing values (MLMV) estimation. We explored whether the effects of ADHD‐PRS on executive function were mediated by [^18^F]FDG‐PET metabolism, CSF Aβ1‐42, or CSF p‐tau181. SEM is preferred for multiple mediators, since it accounts for their correlation.^24^ In addition, correlations between residuals of the mediators were included in the model to improve statistical efficiency.^24^ We used the Stata command *medsem* to estimate the indirect effects using the Monte Carlo approach.^25^ Mediation was inferred only in the presence of significant indirect effects.^26^

Statistical analyses were conducted using Stata version 14.0 (StataCorp, College Station, TX, USA). A two‐sided *p*‐value lower than .05 was considered statistically significant. The voxel‐wise linear regression analyses were conducted within RStudio, utilizing R version 4.1.2 and the RMINC package version 1.5.3.0. We used random field theory (RFT) to correct brain imaging results for multiple comparisons.

## 3 RESULTS

We studied a total of 938 participants with cognitive impairment (674 MCI and 264 AD). The mean (SD) age of the sample was 73.5 (7.5) years, of which 383 (40.8%) were women, and all participants self‐identified as White (935 as not Hispanic/Latino). Table 1 shows the participant's demographic and biomarker characteristics.

**TABLE 1:** Demographic and clinical characteristics of the population.

	Overall (*N* = 938)	MCI (*N* = 674)	AD (*N* = 264)
Age, y, mean (SD)	73.5 (7.4)	72.9 (7.3)	75 (7.6)
Sex, no. (%)			
Female	383 (40.8)	269 (39.9)	114 (43.2)
Male	555 (59.2)	405 (60.1)	150 (56.8)
Race, no. (%)			
White	938	674	264
Ethnicity, no. (%)			
Not Hispanic/Latino	935 (99.7)	673 (99.9)	262 (99.2)
Unknown	3 (0.3)	1 (0.1)	2 (0.8)
Years of education, mean (SD)	15.7 (2.8)	15.9 (2.8)	15.2 (2.9)
VRFs, mean (SD)^a^	1.7 (1.2)	1.7 (1.2)	1.7 (1.2)
BMI, mean (SD)^b^	26.5 (4.5)	26.8 (4.5)	25.8 (4.5)
CSF Aβ1‐42, pg/mL, mean (SD)^c^	864.9 (422.7)	941.2 (435.9)	668.9 (311.1)
CSF p‐tau181, pg/mL, mean (SD)^c^	30.8 (15.4)	28.6 (14.9)	36.6 (15.3)

Abbreviations: Aβ, amyloid beta; AD; Alzheimer's disease; BMI, body mass index; CSF, cerebrospinal fluid; MCI, mild cognitive impairment; SD, standard deviation; VRFs, vascular risk factors; y, years.

^a^ Represents the sum of VRFs. A total of 937 individuals (674 MCI and 263 AD).

^b^ A total of 937 individuals (673 MCI and 264 AD).

^c^ A total of 696 individuals (501 MCI and 195 AD).

### 3.1 ADHD‐PRS is associated with executive function deficit in individuals with MCI or AD dementia

**FIGURE 1:**
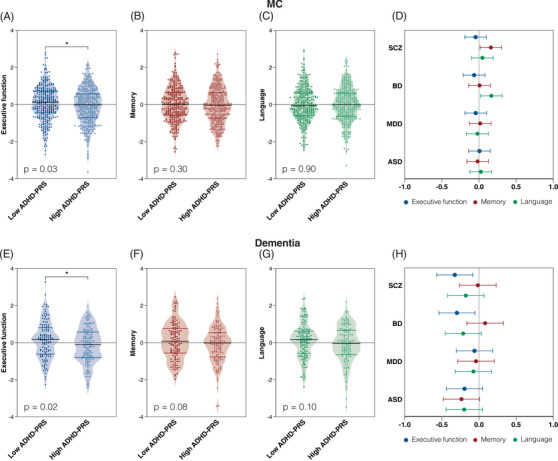
Higher ADHD‐PRS is associated with worsening executive function, but not memory, in individuals with MCI and dementia. Executive function (A, E), memory (B, F), and language (C, G) in individuals (MCI and dementia) dichotomized into low or high ADHD‐PRS groups (*z*‐scores <0 and >0, respectively). Dots show partial residual plots of executive function, memory, or language adjusting for sex, age, and ancestry. Figures (D) and (H) show the β coefficients and 95% confidence intervals of linear regression models assessing executive function, memory, and language function between individuals dichotomized into low or high PRS groups (SCZ‐PRS, BD‐PRS, MDD‐PRS, and ASD‐PRS). Comparisons between groups were performed using linear regression models adjusting for sex, age, and ancestry (using the first seven principal components). ADHD‐PRS, attention‐deficit/hyperactivity disorder polygenic risk score; ASD, autism spectrum disorder; BD, bipolar disorder; MCI, mild cognitive impairment; MDD, major depressive disorder; SCZ, schizophrenia.

In MCI, we observed lower executive function in individuals with high ADHD‐PRS compared to those with low ADHD‐PRS (β = −0.15, 95% CI = −0.30 to −0.008, *p*‐value = .03; Figure 1A). No significant differences in memory performance (β = −0.07, 95% CI = −0.22 to 0.07, *p*‐value = .30; Figure 1B) or language function (β = −0.009, 95% CI = −0.15 to 0.13, *p*‐value = .90; Figure 1C) were observed between high and low ADHD‐PRS. Higher memory function was observed in MCI individuals with high SCZ‐PRS when compared to low SCZ‐PRS (β = 0.16, 95% CI = 0.01 to 0.30, *p*‐value = .03; Figure 1D). In addition, a higher language function was observed in individuals with high BD‐PRS when compared to low BD‐PRS (β = 0.16, 95% CI = 0.02 to 0.31, *p*‐value = .02; Figure 1D).

In AD dementia, we observed lower executive function in individuals with high ADHD‐PRS compared to those with low ADHD‐PRS (β = −0.28, 95% CI = −0.52 to −0.03, *p*‐value = .02; Figure 1E). No significant differences in memory performance were observed between high and low ADHD‐PRS (β = −0.21, 95% CI = −0.45 to 0.02, *p*‐value = .08; Figure 1F). Similarly, no significant differences in language function were observed between high and low ADHD‐PRS (β = −0.20, 95% CI = −0.44 to 0.04, *p*‐value = .10; Figure 1G). Lower executive function was observed in individuals with high SCZ‐PRS when compared to low SCZ‐PRS (β = −0.32, 95% CI = ‐0.56 to −0.08, *p*‐value = .009; Figure 1H), and in individuals with high BD‐PRS when compared to low BD‐PRS (β = −0.29, 95% CI = −0.53 to −0.05, *p*‐value = .01; Figure 1H).

Differences in executive function between high and low ADHD‐PRS remained significant after adjusting for AD‐PRS and VRFs (Table S2). For AD dementia participants, results remained significant after adjusting for years of education and BMI, whereas for MCI participants the findings were non‐significant (Table S2).

### 3.2 ADHD‐PRS is associated with higher levels of CSF p‐tau181 in individuals with AD dementia

**FIGURE 2:**
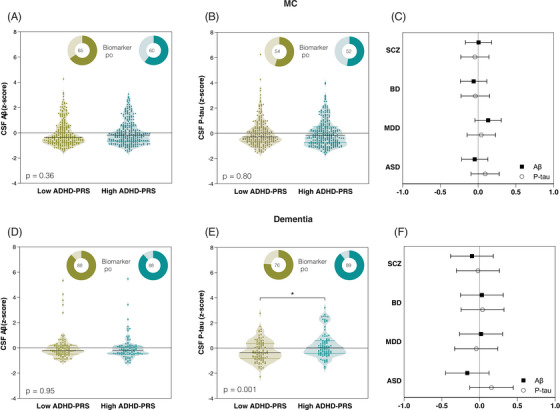
Higher ADHD‐PRS is associated with higher levels of CSF p‐tau in individuals with dementia. CSF levels of Aβ1‐42 (A, D) and p‐tau181 (B, E) in individuals (MCI and dementia) dichotomized into low or high ADHD‐PRS groups (*z*‐scores <0 and >0, respectively). Dots show partial residual plots of CSF biomarkers adjusting for sex, age, and ancestry. Dark colors in donut charts represented the percentage of individuals positive for the biomarker. Figures (C) and (F) show the β coefficients and 95% confidence intervals of linear regression models assessing CSF Aβ1‐42 and p‐tau181 between individuals dichotomized into low or high PRS groups (SCZ‐PRS, BD‐PRS, MDD‐PRS, and ASD‐PRS). Comparisons between groups were performed using linear regression models adjusting for sex, age, and ancestry (using the first seven principal components). ADHD‐PRS, attention‐deficit/hyperactivity disorder polygenic risk score; ASD, autism spectrum disorder; Aβ, amyloid beta; BD, bipolar disorder; CSF, cerebrospinal fluid; MCI, mild cognitive impairment; MDD, major depressive disorder; p‐tau181, tau phosphorylated at threonine 181; SCZ, schizophrenia.

No differences in levels of CSF Aβ1‐42 (β = 0.08, 95% CI = −0.09 to 0.25, *p*‐value = .36; Figure 2A) or p‐tau181 (β = −0.02, 95% CI = −0.21 to 0.16, *p*‐value = .80; Figure 2B) were observed within the MCI population when comparing individuals with low and high ADHD‐PRS. There were no differences in CSF Aβ1‐42 or p‐tau181 levels between individuals with high and low PRS for SCZ, BD, MDD, or ASD (Figure 2C).

No differences in levels of CSF Aβ1‐42 (β = −0.007, 95% CI = −0.29 to 0.27, *p*‐value = .95; Figure 2D) were observed within the AD dementia participants. However, we observed higher levels of CSF p‐tau181 (β = 0.48, 95% CI = 0.20 to 0.75, *p*‐value = .001; Figure 2E) in AD dementia participants with higher ADHD‐PRS compared to those with lower ADHD‐PRS. In addition, no differences in CSF Aβ1‐42 or p‐tau181 levels between individuals with high and low PRS for SCZ, BD, MDD, or ASD were observed (Figure 2F). A summary of findings from the PRS analyses can be found in Table S3.

### 3.3 ADHD‐PRS is associated with brain hypometabolism in individuals with AD dementia

**FIGURE 3:**
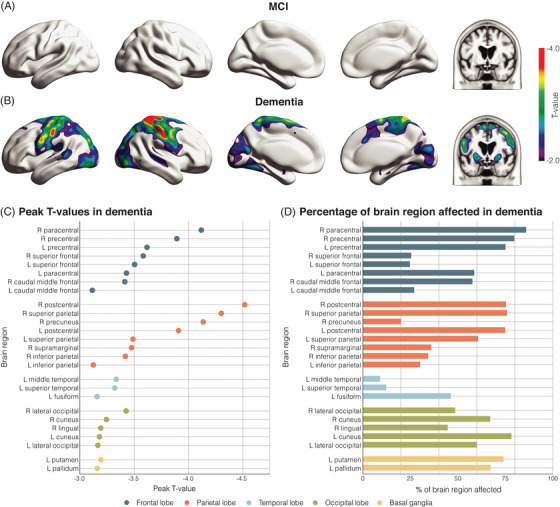
Higher ADHD‐PRS is associated with brain hypometabolism in individuals with dementia. (A, B) Voxel‐wise regression analysis showing the association between ADHD‐PRS and [^18^F]FDG‐PET SUVr. Linear regression models were corrected for multiple comparisons using RFT and adjusted for sex, age, and ancestry (using the first seven principal components). *T*‐values >3.11 survived correction for multiple comparisons. (C) Peak *T*‐values in each brain region identified in the previous step for AD participants. (D) Percentage of each brain region affected in dementia participants, as identified in the previous step. [18F]FDG‐PET, fluorodeoxyglucose positron emission tomography; AD, Alzheimer's disease; ADHD‐PRS, attention‐deficit/hyperactivity disorder polygenic risk score; MCI, mild cognitive impairment; RFT, random field theory; SUVr, standardized uptake value ratio.

In MCI participants, there was no significant association between ADHD‐PRS and [^18^F]FDG‐PET SUVr after correcting for multiple comparisons (Figure 3A). In AD dementia individuals, higher ADHD‐PRS was associated with lower [^18^F]FDG‐PET SUVr after correcting for multiple comparisons in the frontal, parietal, temporal, and occipital regions (Figure 3B), as well as regions from the basal ganglia (Figure 3B). Peak *t*‐values in AD dementia participants and the percentage of brain regions affected in the voxel‐wise analyses are presented in Figure 3C and 3D, respectively.

### 3.4 Brain hypometabolism mediates ADHD‐PRS effects on executive dysfunction

**FIGURE 4:**
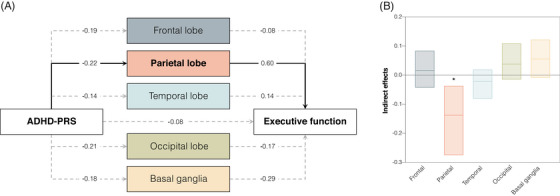
Glucose hypometabolism in the parietal lobe mediates the effects of ADHD‐PRS on executive function in individuals with dementia. (A) Structural equation model testing the mediating effects of brain metabolism in the frontal lobe, parietal lobe, temporal lobe, occipital lobe, and basal ganglia in the association between ADHD‐PRS and executive function. [^18^F]FDG‐PET SUVr values were extracted from regions previously associated with ADHD‐PRS in the voxel‐wise analysis of dementia individuals and included as mediators. A weighted average was calculated for each major brain region (frontal lobe, parietal lobe, temporal lobe, occipital lobe, and basal ganglia). The numbers presented in the figure are standardized β estimates from the structural equation model. Solid lines represent statistically significant indirect effects. All associations were adjusted for age, sex, and ancestry (using the first seven principal components). Brain metabolism was measured with [^18^F]FDG‐PET SUVr. (B) Bars represent means and 95% CIs of indirect effects from each mediator. ADHD‐PRS, attention‐deficit/hyperactivity disorder polygenic risk score; CI, confidence interval; [^18^F]FDG‐PET, fluorodeoxyglucose positron emission tomography; SUVr, standardized uptake value ratio.

We used SEM to investigate whether [^18^F]FDG‐PET SUVr mediated the observed effects of ADHD‐PRS on executive function in AD. As mediators, we included [^18^F]FDG‐PET SUVr values derived from brain regions linked to ADHD‐PRS (Figure 3B–D). We combined SUVR values extracted from regions located within the frontal, parietal, temporal, and occipital lobes and the basal ganglia. To do this, we calculated a weighted average using both hemispheres. Analyses showed statistically significant indirect effects for the parietal lobe (Figure 4A–B), indicating that lower [^18^F]FDG‐PET SUVr within this region mediated the association between ADHD‐PRS and executive function (Figure 4A). Approximately 63% of the effects of ADHD‐PRS on executive function were mediated by [^18^F]FDG‐PET SUVr in the parietal lobe. Indirect effects and corresponding 95% confidence intervals (CIs) are depicted in Figure 4B. The analyses using CSF Aβ1‐42 or CSF p‐tau181 as mediator did not show significant indirect effects, suggesting an absence of mediation (Figure S1a and S1b, respectively).

## 4 DISCUSSION

This study aimed to determine whether the ADHD‐PRS was linked to cognitive dysfunction and higher biomarker levels of AD pathology in individuals with MCI and dementia. We showed that higher ADHD‐PRS was associated with consistent deficits in executive function, but not memory or language. In addition, individuals with AD dementia with higher ADHD‐PRS showed higher levels of tau pathology, along with glucose hypometabolism, across widespread cortical and subcortical brain regions, with the frontal and parietal lobes being predominantly affected. Of interest, there were no significant associations between the genetic risk for other prevalent psychiatric disorders (SCZ, BD, MDD, or ASD) and AD pathology. Finally, the association between higher ADHD‐PRS and lower executive function was mediated by lower glucose metabolism.

Our results support that the genetic risk for ADHD, measured with ADHD‐PRS, is associated with worse executive function in individuals with cognitive impairment. A substantial body of literature has consistently documented executive function deficits in individuals with ADHD.^27^ In fact, prior studies have proposed that the cognitive deficits observed in ADHD might be clinically indistinguishable from those found in MCI,^28^ with comparable performance on executive function tests.^29^ Our findings imply that in individuals with MCI or dementia due to AD, a higher genetic risk for ADHD is associated with executive function deficits that surpass what is typically anticipated for these conditions. These findings hold substantial clinical relevance, given that reduced executive function in AD has been linked to impairments in everyday functioning,^30^ heightened neuropsychiatric symptoms,^31^ and lower quality of life.^32^ No consistent associations were observed for other prevalent psychiatric disorders. In this context, our results suggest that the coexistence of ADHD and AD might contribute to a more severe disease manifestation characterized by worsening cognitive function, thereby highlighting the potential need for a tailored assessment for this population.

The ADHD‐PRS was associated with higher CSF p‐tau181, but not Aβ1‐42, in individuals with AD dementia. This is in accordance with data showing that higher genetic risk for ADHD was associated with longitudinal increases in CSF p‐tau among cognitively unimpaired individuals.^13^ CSF p‐tau levels have been shown to correlate with the spread of tau pathology in the cortex according to the staging system proposed by Braak.^33^, ^34^ The accumulation of tau tangles, in turn, is associated with greater brain glucose hypometabolism ^35^ and atrophy,^36^ as well as cognitive impairment.^35^ Notably, no associations were observed between the PRS for SCZ, BD, MDD, or ASD with biomarkers of Aβ or tau pathology, indicating that higher levels of tau are specifically associated with the ADHD‐PRS. In summary, these results support the notion that the genetic risk for ADHD is associated with more severe AD‐related tau pathology.

The ADHD‐PRS was associated with lower glucose metabolism across widespread cortical and subcortical regions. The results were more pronounced in individuals with dementia, in whom more pronounced hypometabolism is expected.^11^ Regions of the frontal and parietal lobes were predominantly affected. The involvement of cortical and subcortical structures was not surprising given prior functional MRI (fMRI) studies indicating decreased brain activation in fronto‐striato‐parietal circuits in individuals with ADHD.^37^ In addition, atrophy in the parietal and frontal cortices has been demonstrated in middle‐aged ^38^ and older adults ^39^ with ADHD. Notably, lower glucose metabolism measured with [^18^F]FDG‐PET has been reported in adults with histories of hyperactivity of childhood onset.^40^ Overall, our findings support that higher ADHD‐PRS predisposes to more pronounced neurodegeneration across widespread brain regions in AD, with frontal and parietal regions predominantly affected.

The effects of ADHD on executive function were mediated by lower glucose metabolism in regions of the parietal lobe, suggesting a possible underlying mechanistic explanation for our clinical findings. Both the frontal and parietal lobes have been implicated repeatedly in executive function performance as part of the fronto‐parietal attention network.^41^ In ADHD, fMRI studies showed functional abnormalities in parietal brain regions during attention tasks.^37^ In AD, individuals with a dysexecutive presentation exhibited higher rates of tau accumulation^42^ and glucose hypometabolism^43^ in the frontal and parietal brain regions. Altogether our findings suggest that parietal neurodegeneration may contribute to ADHD‐related executive impairment in patients with underlying AD.

It is necessary to acknowledge the inherent limitations of this study. The sample analyzed was not subjected to a detailed clinical assessment for ADHD diagnosis. Therefore, the genetic risk for ADHD may identify asymptomatic older adults with genetic susceptibility to ADHD and/or individuals with undiagnosed ADHD. Although ADHD‐PRS has been shown to be associated with symptoms and diagnosis of ADHD, contributing environmental factors are relevant for the manifestation of the disease.^1^ Individuals with a prior history of SCZ, MDD, or BD are not included in the ADNI cohort. Therefore, participants analyzed in our study are more likely to have a lower overall genetic predisposition to these disorders when compared to the general population. To address this potential bias, future studies should aim to replicate our findings using data collected from population‐based samples that include more individuals with co‐occurring psychiatric disorders. Finally, our study population consisted almost exclusively of White participants. Consequently, future studies should prioritize the inclusion of more diverse populations.

In conclusion, our results suggest that older participants with MCI and dementia due to AD and higher ADHD‐PRS have a more severe presentation of the disease.

## CONFLICT OF INTEREST STATEMENT

J.T. reported consulting fees from the Neurotorium educational platform, Alzheon Inc. V.L.V. reported consulting fees from Life Molecular Imaging, payment or honoraria for lectures, presentations, speakers bureaus, manuscript writing, or educational events from AC Immune, Eli Lilly, and Life Molecular Imaging. P.R.N. reported consulting fees from Novonordisk and Eisai, payment or honoraria for lectures, presentations, speakers bureaus, manuscript writing, or educational events from NovoNordisk; and participation on a Data Safety Monitoring Board or Advisory Board from Novonordisk and Eisai. E.R.Z. reported participation on a Data Safety Monitoring Board or Advisory Board from Novo Nordisk, Nintx and Masima. ERZ is a co‐founder and minority shareholder in Masima. E.R.Z. reported grants or contracts from the Alzheimer’s Association, National Academy of Neuropsychology, CAPES, CNPQ, Instituto Serrapilheira, FAPERGS, Brazilian Ministry of Health, Secretary of Health of Rio Grande do Sul and FAPESP. T.K.K. reported grants or contracts from the National Institutes of Health (NIH), Aina (Ann) Wallströms and Mary‐Ann Sjöbloms stiftelsen, Emil och Wera Cornells stiftelsen, Swedish Research Council, Alzheimer's Association, Swedish Alzheimer Foundation, consulting fees from Quanterix, payment or honoraria for lectures, presentations, speakers bureaus, manuscript writing, or educational events from University of Wisconsin‐Madison, University of Pennsylvania, NIH, Advent Health, CQDM Canada, Icahn School of Medicine at Mount Sinai, NY, USA. O.L.L. reported participation on a Data Safety Monitoring Board from Acumen. L.A.R. has received grant or research support from, served as a consultant to, and served on the speakers’ bureau of Abdi Ibrahim, Abbott, Aché, Adium, Apsen, Bial, Cellera, EMS, Hypera Pharma, Knight Therapeutics, Libbs, Medice, Novartis/Sandoz, Pfizer/Upjohn/Viatris, Shire/Takeda, and Torrent in the last three years. The ADHD and Juvenile Bipolar Disorder Outpatient Programs chaired by L.A.R. have received unrestricted educational and research support from the following pharmaceutical companies in the last three years: Novartis/Sandoz and Shire/Takeda. L.A.R. has received authorship royalties from Oxford Press and ArtMed. All other authors report no conflicts of interest. Author disclosures are available in the Supporting Information.

## CONSENT STATEMENT

The institutional review boards of all participating sites approved the Alzheimer's Disease Neuroimaging Initiative (ADNI) study, and all research participants or their authorized representatives provided written informed consent.

## Supporting information



Supporting Information

Supporting Information
