# Correction to “Impact of the polygenic risk scores for attention‐deficit/hyperactivity disorder in Alzheimer's disease”

**DOI:** 10.1002/alz.70467

**Published:** 2025-07-19

Leffa DT, Povala G, Bellaver B, et al. Impact of the polygenic risk scores for attention‐deficit/hyperactivity disorder in Alzheimer's disease. *Alzheimers Dement*. 2025;21(2):e70003. https://doi.org/10.1002/alz.70003

In Figures 1 and 2, “MC” is displayed instead of “MCI.” Additionally, Figure 2 is missing the percentage symbols and includes a truncated label “Biomarker po” instead of “Biomarker positivity.”

We apologize for this error.

