# Supplementary material for: Impact of the polygenic risk scores for attention‐deficit/hyperactivity disorder in Alzheimer's disease
Source: Alzheimers Dement. 2025 Feb 25;21(2):e70003. doi: 10.1002/alz.70003 (PMC11853731; doi:10.1002/alz.70003)
Supplement: Supplementary file 1 — Supporting Information [file ALZ-21-e70003-s001.docx]

**SUPPLEMENTARY MATERIAL: TABLE OF CONTENTS**

[Supplementary methods 2](#_Toc185578610)

[Polygenic risk scores 2](#_Toc185578611)

[Cognitive composites 3](#_Toc185578612)

[CSF biomarker 4](#_Toc185578613)

[Vascular burden 5](#_Toc185578614)

[Supplementary Table 1. Baseline demographic and clinical characteristics of individuals with and without CSF data. 6](#_Toc185578615)

[Supplementary Table 2. Sensitivity analyses of the association between ADHD-PRS and executive function. 7](#_Toc185578616)

[Supplementary Table 3. Summary of findings from PRS analyses. 8](#_Toc185578617)

[Supplementary Figure 1. Mediation analyses using CSF biomarkers of AD. 11](#_Toc185578618)

[References 12](#_Toc185578619)

# Supplementary methods

## Polygenic risk scores

Data were available across three genotyping platforms: (1) the Human610-Quad platform, (2) the HumanOmniExpress, and (3) Omni 2.5 M platform. Imputation and merging of the different platforms were performed as previously described ^1^. We calculated PRSs from the genome wide association studies of ADHD ^2^, AD ^3^, major depression ^4^, bipolar disorder ^5^, schizophrenia ^6^, and autism spectrum disorder ^7^. PRSs were calculated using the PRSice software v2.2 ^8^. Independent single nucleotide polymorphisms (SNPs) were classified based on a 250-kb window and 0.1 r2 linkage disequilibrium criteria. After applying the quality control filters, 212,846 variants were retained for PRS analysis. PRS was calculated using the additive model, which is the weighted sum of risk alleles. Eleven PRSs were calculated for each condition using subset of SNPs selected according to the following GWAS p-value thresholds: 1, 0.5, 0.4, 0.3, 0.2, 0.1, 0.05, 0.005, 0.0005, 5e-6, and 5e-8. Then, we extracted the first principal component for each PRS across all P-value thresholds. This approach has been empirically validated as effective and robust to quantify polygenic risk, reducing multiple testing and type I error ^9^. PRSs were transformed into z-scores for better visualization. To investigate populational structure, principal components analysis was conducted using PLINK 1.9 ^10^. We retained seven principal components to account for any ancestry differences in genetic structure that could bias the results, as previously done for ADNI datasets ^1^.

## Cognitive composites

Cognitive function (executive function, memory, and language) was assessed using cognitive composite scores validated for the ADNI sample ^11-13^. The executive function composite score included the following: Digit Symbol Substitution Test; Digit Span Forward and Backward; Trail Making Test A and B; Clock Drawing Test; Alzheimer’s Disease Assessment Schedule-Cognitive Subscale (ADAS-Cog) Number Cancellation; Montreal Cognitive Assessment (MoCA) Alternating Trail Making, Digit Span Forward, Digit Span Backward, Letters and Tapping, Serial 7s, and Abstraction. The memory composite score included: Rey Auditory Verbal Learning Test (RAVLT); ADAS-Cog Word Recall and Word Recognition; Mini-Mental State Examination (MMSE) Delayed Recall; and Logical Memory Test I (Immediate Recall) and II (Delayed Recall). The language composite score included: Category Fluency Test Animal and Vegetable; Boston Naming Test; MMSE Command, Repetition, Reading, Writing; ADAS-Cog Commands, Naming Task, Ideational Praxis; MoCA Naming, Sentence Repetition, and Verbal Fluency.

## CSF biomarker

CSF biomarker measurements outside the analytical range (<200 pg/mL or >1700 pg/mL for Aβ_1-42_; <8 pg/mL or >120pg/mL for p-tau_181_) were handled as previously recommended ^14^. For statistical analyses, they were set to the lower and upper detection limit. For figures, values outside the analytical range were estimated from the original signals based on extrapolated calibration curves. For Aβ_1-42_, 77 values were >1700 pg/mL and none were <200 pg/mL. For p-tau_181_, one value was >120 pg/mL and one was <8 pg/mL.

## Vascular burden

Vascular risk factors (VRFs) were assessed using a composite score, as previously described ^15^. Briefly, we calculated the score based on the presence or absence of the following conditions: (1) cardiovascular disease [coronary artery disease (myocardial infarction, angina, stent placement, angioplasty, coronary artery bypass graft, coronary insufficiency), heart failure, or intermittent claudication]; (2) hypertension; (3) diabetes mellitus; (4) hyperlipidemia; (5) stroke or transient ischemic attack (TIA); (6) smoking (ever or never); (7) atrial fibrillation; and (8) left ventricular hypertrophy. The score was obtained as the sum of VRFs (ranging from zero to eight). An elevated VRF burden was defined as a vascular composite score equal to or higher than two ^15^.

## Supplementary Table 1. Baseline demographic and clinical characteristics of individuals with and without CSF data.

|  | **Overall**  **(N = 938)** | **CSF data**  **(N = 696)** | **No CSF data**  **(N = 242)** |
| --- | --- | --- | --- |
| Age, y, mean (SD) | 73.5 (7.4) | 73.01 (7.6) | 74.9 (6.9) |
| Sex, No. (%) |  |  |  |
| Female | 383 (40.8) | 278 (39.9) | 105 (43.4) |
| Male | 555 (59.2) | 418 (60.1) | 137 (56.6) |
| Race, No. (%) |  |  |  |
| White | 938 | 696 | 242 |
| Ethnicity, No. (%) |  |  |  |
| Not Hispanic/Latino | 935 (99.7) | 695 (99.9) | 240 (99.2) |
| Unknown | 3 (0.3) | 1 (0.1) | 2 (0.8) |
| Years of education, mean (SD) | 15.7 (2.8) | 15.9 (2.8) | 15.2 (2.9) |
| VRFs, mean (SD)* | 1.7 (1.2) | 1.6 (1.2) | 1.8 (1.3) |
| BMI, mean (SD)** | 26.5 (4.5) | 26.6 (4.5) | 26.2 (4.6) |

Abbreviations: SD, standard deviation; y, years; VRFs, vascular risk factors; BMI, body mass index.

* Represents the sum of VRFs. Total of 937 individuals (696 with CSF data and 241 without it)

** Total of 937 individuals (695 with CSF data and 242 without it)

## Supplementary Table 2. Sensitivity analyses of the association between ADHD-PRS and executive function.

| Controlling for: | β (95% CI) | *p*-value |
| --- | --- | --- |
|  | MCI | |
| AD-PRS | -.15 (-.30, -.01) | .03 |
| VRFs | -.14 (-.29, -.002) | .04 |
| BMI | -.13 (-.28, .006) | .06 |
| Years of study | -.11 (-.25, .02) | .11 |
|  | AD | |
| AD-PRS | -.28 (-.52, -.04) | .02 |
| VRFs | -.27 (-.51, -.03) | .02 |
| BMI | -.28 (-.52, -.03) | .02 |
| Years of study | -.26 (-.51, -.02) | .03 |

Supplementary Table 2 shows the association between ADHD-PRS and executive function controlling for potential confounders. β and p-values were obtained from a linear regression model assessing the effects of ADHD-PRS on executive function adjusting for sex, age, ancestry (using the first seven principal components), plus an additional confounder included in the first column.

Abbreviations: ADHD-PRS, attention-deficit/hyperactivity disorder polygenic risk score; MCI, mild cognitive impairment; AD, Alzheimer’s disease; AD-PRS, Alzheimer’s disease polygenic risk score; VRFs, vascular risk factors; BMI, body mass index.

## Supplementary Table 3. Summary of findings from PRS analyses.

| **Population** | **PRS** | **Executive function** | **Memory** | **Language** | **CSF Aβ_1-42_** | **CSF p-tau_181_** |
| --- | --- | --- | --- | --- | --- | --- |
| MCI | ADHD | **β=-0.15, 95% CI=-0.30 to -0.008, p=0.03** | β=-0.07, 95% CI=-0.22 to 0.07, p=0.30 | β=-0.009, 95% CI=-0.15 to 0.13, p=0.90 | β=0.08, 95% CI=-0.09 to 0.25, p=0.36 | β=-0.02, 95% CI=-0.21 to 0.16, p=0.80 |
|  | SCZ | β=-0.04, 95% CI=-0.19 to 0.10, p=0.55 | **β=0.16, 95% CI=0.01 to 0.30, p=0.03** | β=0.04, 95% CI=-0.09 to 0.19, p=0.53 | β=0.006, 95% CI=-0.16 to 0.18, p=0.94 | β=-0.04, 95% CI=-0.22 to 0.14, p=0.66 |
|  | BD | β=-0.06, 95% CI=-0.21 to 0.08, p=0.38 | β=0.009, 95% CI=-0.13 to 0.15, p=0.90 | **β=0.16, 95% CI=0.02 to 0.31, p=0.02** | β=-0.05, 95% CI=-0.23 to 0.11, p=0.51 | β=-0.03, 95% CI=-0.22 to 0.15, p=0.70 |
|  | MDD | β=-0.04, 95% CI=-0.18 to 0.10, p=0.56 | β=0.01, 95% CI=-0.13 to 0.16, p=0.82 | β=-0.02, 95% CI=-0.16 to 0.12, p=0.78 | β=0.13, 95% CI=-0.04 to 0.30, p=0.13 | β=0.04, 95% CI=-0.14 to 0.23, p=0.66 |
|  | ASD | β=0.006, 95% CI=-0.13 to 0.15, p=0.93 | β=-0.01, 95% CI=-0.16 to 0.12, p=0.82 | β=0.02, 95% CI=-0.12 to 0.17, p=0.73 | β=-0.04, 95% CI=-0.21 to 0.13, p=0.62 | β=0.09, 95% CI=-0.09 to 0.28, p=0.32 |
| Dementia | ADHD | **β=-0.28, 95% CI=-0.52 to -0.03, p=0.02** | β=-0.21, 95% CI=-0.45 to 0.02, p=0.08 | β=-0.20, 95% CI=-0.44 to 0.04, p=0.10 | β=-0.007, 95% CI=-0.29 to 0.27, p=0.95 | **β=0.48, 95% CI=0.20 to 0.75, p=0.001** |
|  | SCZ | β=-0.32, 95% CI=-**0.56 to -0.08, p=0.009** | β=-0.01, 95% CI=-0.26 to 0.23, p=0.90 | β=-0.17, 95% CI=-0.42 to 0.06, p=0.16 | β=-0.10, 95% CI=-0.38 to 0.18, p=0.48 | β=-0.02, 95% CI=-0.30 to 0.26, p=0.88 |
|  | BD | **β=-0.29, 95% CI=-0.53 to -0.05, p=0.01** | β=0.08, 95% CI=-0.16 to 0.33, p=0.50 | β=-0.21, 95% CI=-0.45 to 0.03, p=0.09 | β=0.02, 95% CI=-0.25 to 0.31, p=0.84 | β=0.03, 95% CI=-0.24 to 0.32, p=0.79 |
|  | MDD | β=-0.06, 95% CI=-0.30 to 0.18, p=0.63 | β=-0.04, 95% CI=-0.28 to 0.20, p=0.75 | β=-0.07, 95% CI=-0.31 to 0.17, p=0.55 | β=0.01, 95% CI=-0.26 to 0.30, p=0.89 | β=-0.04, 95% CI=-0.33 to 0.24, p=0.76 |
|  | ASD | β=-0.19, 95% CI=-0.43 to 0.05, p=0.12 | β=-0.23, 95% CI=-0.48 to 0.01, p=0.06 | β=-0.19, 95% CI=-0.44 to 0.04, p=0.11 | β=-0.16, 95% CI=-0.45 to 0.12, p=0.27 | β=0.15, 95% CI=-0.13 to 0.44, p=0.28 |

Supplementary Table 3 shows a summary of the associations between PRS (ADHD, SCZ, BD, MDD, and ASD) and cognition (executive function, memory, language), as well as PRS and CSF biomarkers (Aβ_1-42_ and CSF p-tau_181_). β, 95% CI and p-values were obtained from a linear regression model assessing the effects of PRS (transformed into z-scores and categorized into low and high [<0 and >0, respectively]) on cognition or CSF biomarkers adjusting for sex assigned at birth, age, and ancestry (using the first seven principal components).

Abbreviations: MCI, mild cognitive impairment; CSF, cerebrospinal fluid; Aβ_,_ amyloid-β; p-tau_181_, tau phosphorylated at threonine 181; PRS, polygenic risk score; ADHD, attention-deficit/hyperactivity disorder; SCZ, schizophrenia; BD, bipolar disorder; MDD, major depressive disorder; ASD, autism spectrum disorder.

## Supplementary Figure 1. Mediation analyses using CSF biomarkers of AD.


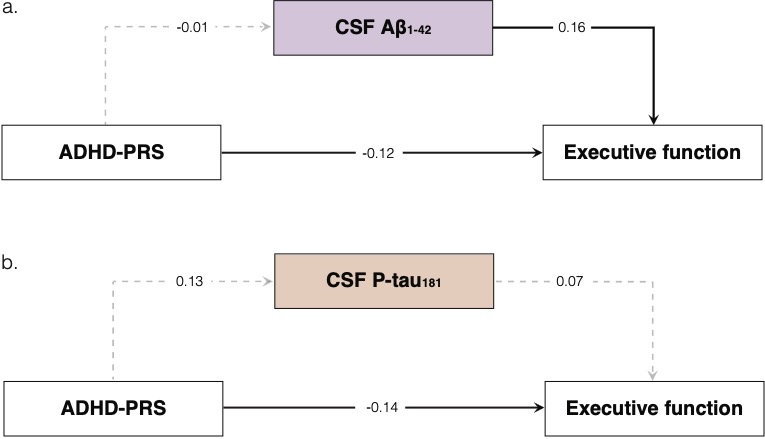


Structural equation model testing the mediating effects of CSF Aβ_1-42_ (a) and p-tau_181_ (b) in the association between ADHD-PRS and executive function in individuals with dementia. The numbers presented in the figure are standardized β estimates from the structural equation model. Solid lines represent statistically significant effects. All associations were adjusted for age, sex, and ancestry (using the first seven principal components).

Abbreviations: ADHD-PRS, attention-deficit/hyperactivity disorder polygenic risk score. AD, Alzheimer’s disease.

# References

1. Zettergren A, Lord J, Ashton NJ, et al. Association between polygenic risk score of Alzheimer's disease and plasma phosphorylated tau in individuals from the Alzheimer's Disease Neuroimaging Initiative. *Alzheimers Res Ther*. Jan 8 2021;13(1):17. doi:10.1186/s13195-020-00754-8

2. Demontis D, Walters RK, Martin J, et al. Discovery of the first genome-wide significant risk loci for attention deficit/hyperactivity disorder. *Nat Genet*. Jan 2019;51(1):63-75. doi:10.1038/s41588-018-0269-7

3. Jansen IE, Savage JE, Watanabe K, et al. Genome-wide meta-analysis identifies new loci and functional pathways influencing Alzheimer's disease risk. *Nat Genet*. Mar 2019;51(3):404-413. doi:10.1038/s41588-018-0311-9

4. Howard DM, Adams MJ, Clarke T-K, et al. Genome-wide meta-analysis of depression identifies 102 independent variants and highlights the importance of the prefrontal brain regions. *Nature Neuroscience*. 2019/03/01 2019;22(3):343-352. doi:10.1038/s41593-018-0326-7

5. Mullins N, Forstner AJ, O'Connell KS, et al. Genome-wide association study of more than 40,000 bipolar disorder cases provides new insights into the underlying biology. *Nat Genet*. Jun 2021;53(6):817-829. doi:10.1038/s41588-021-00857-4

6. Trubetskoy V, Pardiñas AF, Qi T, et al. Mapping genomic loci implicates genes and synaptic biology in schizophrenia. *Nature*. Apr 2022;604(7906):502-508. doi:10.1038/s41586-022-04434-5

7. Grove J, Ripke S, Als TD, et al. Identification of common genetic risk variants for autism spectrum disorder. *Nature Genetics*. 2019/03/01 2019;51(3):431-444. doi:10.1038/s41588-019-0344-8

8. Euesden J, Lewis CM, O'Reilly PF. PRSice: Polygenic Risk Score software. *Bioinformatics*. May 1 2015;31(9):1466-8. doi:10.1093/bioinformatics/btu848

9. Coombes BJ, Ploner A, Bergen SE, Biernacka JM. A principal component approach to improve association testing with polygenic risk scores. *Genet Epidemiol*. Oct 2020;44(7):676-686. doi:10.1002/gepi.22339

10. Chang CC, Chow CC, Tellier LC, Vattikuti S, Purcell SM, Lee JJ. Second-generation PLINK: rising to the challenge of larger and richer datasets. *GigaScience*. 2015;4(1)doi:10.1186/s13742-015-0047-8

11. Crane PK, Carle A, Gibbons LE, et al. Development and assessment of a composite score for memory in the Alzheimer's Disease Neuroimaging Initiative (ADNI). *Brain Imaging Behav*. Dec 2012;6(4):502-16. doi:10.1007/s11682-012-9186-z

12. Gibbons LE, Carle AC, Mackin RS, et al. A composite score for executive functioning, validated in Alzheimer's Disease Neuroimaging Initiative (ADNI) participants with baseline mild cognitive impairment. *Brain Imaging Behav*. Dec 2012;6(4):517-27. doi:10.1007/s11682-012-9176-1

13. Choi SE, Mukherjee S, Gibbons LE, et al. Development and validation of language and visuospatial composite scores in ADNI. *Alzheimers Dement (N Y)*. 2020;6(1):e12072. doi:10.1002/trc2.12072

14. Hansson O, Seibyl J, Stomrud E, et al. CSF biomarkers of Alzheimer's disease concord with amyloid-β PET and predict clinical progression: A study of fully automated immunoassays in BioFINDER and ADNI cohorts. *Alzheimers Dement*. Nov 2018;14(11):1470-1481. doi:10.1016/j.jalz.2018.01.010

15. Ferrari-Souza JP, Brum WS, Hauschild LA, et al. Vascular risk burden is a key player in the early progression of Alzheimer's disease. *Neurobiol Aging*. Apr 2024;136:88-98. doi:10.1016/j.neurobiolaging.2023.12.008
